# 

**Published:** 1879-01

**Authors:** 


### CONTENTS:

ARTICLE I.—Arsenious Acid in Dentistry. By J. Hardman, D. D. S. .......... 385

ARTICLE II.—Address to the Graduates of the Baltimore College of Dental Surgery. By Rev. Julius E. Grammer, D. D. .......... 395

ARTICLE III.—Alveolo-Dental Abscess. By D. D. Smith, M. D., D. D. S. .......... 404

ARTICLE IV.—Filling Proximal Cavities in Bicuspids and Molars. By J. A. W. Davis .......... 41[illegible]

ARTICLE V.—Amalgam for Tooth Filling .......... 41[illegible]

ARTICLE VI.—A New Elastic Gum .......... 42[illegible]

ARTICLE VII.—Lancing the Gums .......... 42[illegible]

### EDITORIAL, ETC.

Amalgam .......... 424
New Method of Attaching Gold Crowns to Natural Roots of Teeth .......... 425
Uranine.—A New Aniline Color .......... 425
Vick's Floral Guide .......... 426

### MONTHLY SUMMARY.

Diffusion of Carcinoma by the Nerves .......... 427
To Blister the Skin Quickly .......... 428
An Early Symptom of Tabes Dorsalis .......... 428
Deaths from Anaesthetics .......... 429
A New Disinfectant .......... 429
Poisoning by Chlorate of Potash .......... 430
Warning against the Hypodermic use of Morphia .......... 430
Female Physicians .......... 430
Absorption of Lime Salts .......... 431
The Skulls of Women .......... 431
Healing of Pieces Separated from the Human Body .......... 432
Conservative Surgery .......... 4[illegible]
Disadvantages of Salicylic Acid as a Mouth-Wash .......... 4[illegible]
Heavy Sentence of a Dentist .......... 432

